# Combined Modality Treatment Including Methotrexate-Based Chemotherapy For Primary CENTRAL Nervous System Lymphoma: A Single Institution Experience

**DOI:** 10.4084/MJHID.2009.020

**Published:** 2009-12-14

**Authors:** Stefan Hohaus, Luciana Teofili, Mario Balducci, Stefania Manfrida, Angelo Pompucci, Francesco D’Alo’, Giuseppina Massini, Luigi Maria Larocca, Roberto Marra, Sergio Storti

**Affiliations:** 1 Istituto di Ematologia, di Radioterapia; 2 di Neurochirurgia; 3 e di Anatomia PatologicaUniversita’ Cattolica S. Cuore, Rome, Italy

## Abstract

Chemotherapy including high-dose methotrexate (HD-MTX), with or without radiotherapy, is standard treatment for primary central nervous system lymphoma (PCNSL). It remains controversial whether addition of other drugs will add to therapeutic efficacy. We report here on 41 patients with PCNSL treated using a combined treatment modality, including HD-MTX (3.5 g/m^2^ for 2 cycles) prior to whole brain radiotherapy (WBRT). In 22 patients, the chemotherapy was intensified by adding high-dose cytosine arabinoside (HD-AraC) (2g/m^2^ for 4 doses for 2 cycles). Complete remission at the end of the combined treatment was obtained in 23 of 34 assessable patients (67%), and the predicted 5-year overall and disease-free survival rates were 24% and 46%, respectively, without differences between treatment groups. The addition of HD-AraC was complicated by severe infections in 17/22 (77%) patients, resulting in 3 toxic deaths. Our study indicates that addition of HD-AraC may not improve clinical outcome in PCNSL, while it increases toxicity. More targeted and less toxic therapies are warranted.

## Introduction:

Non-Hodgkin lymphomas exclusively localized in the brain are usually B-cell lymphomas of the diffuse large B cell type and are characterized by an aggressive clinical course^1^,^2^. Surgical procedures are essential for histological diagnosis, but resection has no benefit for survival, but is often complicated by neurological sequelae. Irradiation in the form of whole brain radiotherapy (WBRT) can often induce remissions, but the majority of patients relapse, and median overall survival is only 11 to 14 months^3^. The addition of combination chemotherapy such as CHOP, which is standard treatment for systemic NHL, but does not contain drugs which cross the blood brain barrier, to WBRT did not improve the outcome of patients with primary CNS lymphomas (PCNSL)^1^–^3^. Only the combination of methotrexate administered at high doses prior to WBRT has improved response rates and survival in PCNSL^4^,^5^. There is a broad consensus that HD-MTX is the single most important cytotoxic drug in the treatment of PCNSL^6^.

The combined modality of chemotherapy including MTX at high doses followed by WBRT is now considered standard treatment. Despite the progress reported in clinical studies prognosis still remains poor. The International Extranodal Lymphoma study group (IELSG) has recently defined a prognostic score consisting of five parameters to predict prognosis, while the Memorial Sloan-Kettering group identified only age and performance status as the only variables in multivariate analysis^7^,^8^. Older age and poor performance status are often limiting factors to perform aggressive therapies, and these patients are often excluded from clinical trials. This may contribute to the fact that progress in the treatment of PCNSL suggested by clinical trials do not result in changes in the outcome, observed over time in cancer registries or single centers^9^–^11^.

Improvement of treatment results are urgently needed, and one strategy is to add cytotoxic agents to increase efficacy of chemotherapy. It is not yet known whether the addition of other cytotoxic agents adds to the efficacy of HD-MTX. Some alkylating agents as BCNU, thiotepa, busulfane, and procarbazine cross the blood-brain barrier at normal doses, while cytosine arabinoside (AraC) crosses the barrier only at high-doses. There is some evidence from retrospective analyses that HD-AraC added to HD-MTX-based chemotherapy may improve outcome^4^,^5^. We report here on a single center experience in 41 patients with PCNSL treated with a combined modality including HD-MTX prior to WBRT, with addition of HD-AraC in 22 of them.

## Patients and Methods

### Patients:

Forty-one consecutive HIV-negative patients diagnosed with PCNSL between March 1995 and May 2004 were included into this study. The median age was 59 years (range 32–78), 21 patients were females and 20 males. Diagnosis of PCNSL was made by histological examination of a stereotactic biopsy in 25 patients and by partial or total resection in 12 patients. In 4 patients, histology was not attempted due to poor performance status or to the high risks associated with the particular localization of the cerebral lesion, in the deep structures of the brain. These patients had radiological features compatible with lymphoma. Moreover, a clonal B-cell population was detected in CSF in 2 patients, while in further 2 patients the diagnosis was suggested by the marked initial improvement of the disease by corticosteroids. Initial evaluation included radiological examination through magnetic resonance imaging (MRI) and/or computed tomography (CT) scan of the brain and lumbar puncture with CSF examination. Fifteen patients presented a single cerebral lesion and 26 patients multiple lesions. CT scans of chest, abdomen and pelvis and bone marrow histology were performed to exclude systemic localizations. Moreover, data about performance status (according to W.H.O. classification) and clinical symptoms were collected, to calculate prognostic score according to IELSG. Patients characteristics are summarized in Table 1.

### Treatment:

For all patients a combined regimen of 2 cycles of high-dose methotrexate (HD-MTX)-based chemotherapy followed by whole brain radiotherapy was planned: 19 patient received HD-MTX only, at the dose of 3.5 g/m^2^ for two courses on days 1 and 22, 13 patients treated between 1998 and 2002 received a sequential therapy of HD-MTX (same schedule as above) followed by HD-AraC at the dose of 2 g/m^2^ every 12 hours for 2 days and for 2 courses (on days 43 and 64), 9 patients younger than 61 years treated between 2000 and 2002 received a combined regimen of HD-MTX 3.5 g/m^2^ the first day and HD-AraC 2 g/m^2^ every 12 hours on days 2 and 3. Systemic HD-MTX was administered as a 6-hour infusion under vigorous hydration and urine alkalinization and was followed by leucovorin rescue. HD-AraC was given as a 3 hour infusion. The 6 patients with a positive CSF cytology received intrathecal chemotherapy in addition to systemic i.v. chemotherapy. In 36 patients, whole brain radiotherapy (40 Gy) with a boost of 20 Gy to the involved area or residual lesions completed the treatment. Prophylaxis with subcutaneous low molecular weight heparin was given from 1999 on.

### Statistical analysis:

The Fisher’s exact test was used to compare the frequencies of patient’s characteristics according to different groups. The response to treatment was evaluated after chemotherapy and at the end of the treatment (chemo- and radiotherapy). Complete Remission (CR) was defined as the disappearance of all enhancing lesions 12. Partial Remission (PR) was defined as the decrease of more than 50% of the size of lesions with contrast enhancement, while progressive disease (PD) was defined by the increase of existing lesions or appearance of any new lesion.

Overall Survival (OS) was calculated from the diagnosis to death for any causes or last follow up Disease Free Survival (DFS) was calculated from the date documenting a CR at the end of the combined treatment until date of relapse. Survival curves were calculated using the method of Kaplan and Meier. The log-rank test was used to compare OS and DFS according to different patient groups. Differences were considered significant only for p<0.05. Statistical analysis was performed using the Stata 7 program.

## Results

### Treatment response and disease control:

Response to chemotherapy could not be assessed in 4 patients who died during chemotherapy due to toxicity. Of 37 assessable patients, 11 patients (30%) achieved complete response, 8 patients (22%) partial response and 18 patients (49%) showed stable or progressive disease. In particular, CR was observed in 4/18 cases (22%) in the group of patients who received HD-MTX alone and in 7/19 cases (37%) in the group treated with HD-MTX and HD-AraC.

Response at the end of combined treatment was assessable in 34 patients, since 3 pts died during radiation therapy due to toxicity. Twenty-three of these 34 patients (67%) achieved complete remission, without differences between the treatment groups [12/16 (75%) in the HD-MTX alone group and 11/18 (61%) in the HD-MTX/HD-AraC group, respectively] (Table 2). In conclusion, in the HD-MTX group 12 of the initial 19 patients (63%) were alive in CR, while in the HD-MTX/HD-AraC group 11 of the initial 22 patients (50%) were alive in CR.

### Outcome:

Thirty-one of the 41 patients deceased, 16 patients due to progressive disease, and 15 patients for other reasons, mainly treatment-related complications (11 patients) (Table 3). Infections and thromboembolic complications were the major causes of death during the treatment period, while neurologic sequelae of the disease/treatment were the major causes of death in remission after the end of treatment. Two patients died due to secondary malignancies (1 colon cancer, 1 diffuse metastatic cancer).

**Figure 1.**
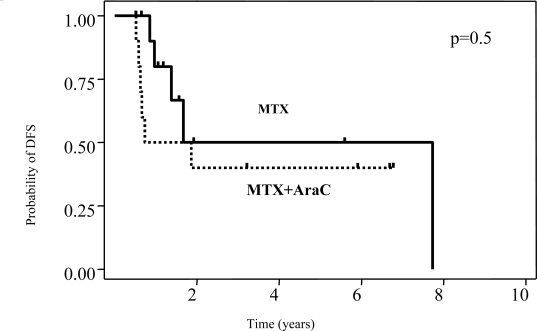
The addition of high-dose cytarabine (AraC) to high-dose methotrexate (MTX) did not improve disease-free survival (DFS). Kaplan-Meier plots for DFS of 23 patients with PCNSL achieving complete remission after combined modality treatment according to type of chemotherapy (MTX alone, n=12; MTX/AraC, n=11) are shown. P refers to log-rank test.

**Figure 2.**
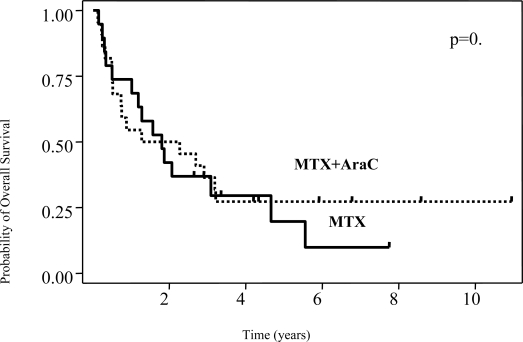
The addition of high-dose cytarabine (AraC) to high-dose methotrexate (MTX) did not improve overall survival. Kaplan-Meier plots for overall survival of 41 patients with PCNSL according to type of chemotherapy (MTX alone, n=19; MTX/AraC, n=22) are shown. P refers to log-rank test.

The predicted 5-year disease-free survival rate was 46% with no differences between treatment groups (p=0.5) (Figure 1). Median overall survival was 22 months, and the predicted 5-year overall survival was 24% (95% C.I.,11–39) without differences between treatment groups (Figure 2). In 35 patients, sufficient data were available to calculate the IELSG prognostic score.

**Figure 3.**
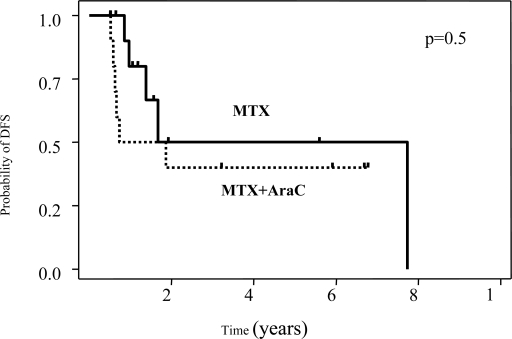
The IELSG prognostic score predicts overall survival in PCNSL. Kaplan-Meier plots for overall survival of 35 patients with PCNSL according to the IELSG score (score 0–1, n=6; score 2–3, n=21, score 4–5, n=8) are shown. P refers to log-rank test.

We observed a trend for differences in overall survival according to the IELSG score (p=0.08) (Figure 3).

### Toxicity:

The HD-MTX-based chemotherapy was complicated by liver and renal toxicity leading to subsequent modification of treatment with early administration of radiotherapy in 4 pts receiving HD-MTX alone (21%) and in 2 pts (10%) of the group receiving HD-MTX/HD-AraC. Haematologic toxicity was frequent and more pronounced in patients receiving additional HD-AraC (Table 2). Grade 4 neutropenia was observed in 7/19 (37%) patients receiving HD-MTX alone, and in 17/22 (77%) patients receiving additional HD-AraC. As a consequence, severe infections particularly occurred in patients receiving HD-AraC and increased the toxic death rate which was particular high among patients receiving the combination of HD-MTX/AraC (3/9, 33%), while the toxic death rate in patients treated with HD-MTX with or without sequential HD-AraC was 8% (1/8) and 16% (3/19) respectively. All 7 toxic death occurred inpatients with an initially reduced performance status (PS ≥ 2). Treatment could be completed as programmed in 15/19 patients (79%) in the group receiving HD-MTX as the only cytotoxic agent, in 9 of 13 patients (69%) in the sequential HD-MTX/HD-AraC and in only 4/9 (44%) patients in the combined HD-MTX/HD-AraC group. Thromboembolic complications were frequent and occurred in 9 of 41 patients (22%): deep venous thrombosis and/or pulmonary embolism were observed at the time of diagnosis in 3 patients, during chemotherapy and radiotherapy in 4 and 2 patients, respectively. Thrombembolic events were fatal. in 3 patients.

## Discussion:

Treatment results in PCNSL are still unsatisfactory due to scarce disease control and to therapy-related toxicity. Although prospective clinical trials indicate a progressive improvement in prognosis for PCNSL in recent years, analysis of cancer registries or single centers experiences do not suggest a significant progress in the treatment results over time^9^–^11^. Combined modality with HD-MTX and WBRT is considered standard, and it is unclear whether the addition of other cytotoxic drugs may improve disease control.

We report a series of 41 patients sequentially treated in a single center, at a median follow-up of 6 years. Focus of our analysis was whether the addition of HD-AraC to a combined approach consisting of HD-MTX and WBRT improves on treatment results.

Retrospective, non-randomized studies indicated that the addition of HD-AraC may improve overall survival. In a retrospective analysis including 226 patients with PCNSL, Blay *et al* found that treatment with chemotherapy versus radiotherapy only, and treatment including HD-MTX or HD-AraC were prognostic factors in the univariate analysis^4^. However, in the multivariate analysis, HD-MTX remained the only independent prognostic factor. The retrospective analysis of 160 patients treated with HD-MTX-based primary chemotherapy by Ferreri *et al* showed that patients treated with combinations of HD-MTX and HD-AraC had a better overall survival than patients treated with HD-MTX only^5^. Therapy with HD-MTX without subsequent radiotherapy is characterized by a high proportion of early relapses after a median of 12–14 months^13^,^14^. Pels *et al* reported on results in 65 patients with PCNSL treated with alternating cycles of chemotherapy including either HD-MTX or HD-AraC and deferred radiotherapy^15^. The median overall survival was 50 months, and results were particularly encouraging in patients younger than 60 years. Six (9%) of the patients died because of treatment-related complications, and systemic toxicity was mainly haematologic. In our series, the addition of HD-AraC combined to HD-MTX increased the risk of infections, resulting in changes of the treatment program in 6 of 22 patients and in toxic death in 3 of 22 patients. In addition, HD-AraC did not increase the complete remission rate, and did not improve disease-free and overall survival. In conclusion, in our hands, the addition of HD-AraC into a combined HD-MTX/WBRT program increased the toxicity of the treatment without improving disease control. As only 4 of the 9 patients in the combined treatment group completed therapy as planned, this might indicate that this therapy is too toxic for the majority of patients, who are often severely debilitated by the disease. A sequential approach appears more feasible. Given however the limited number of patients in our study, it is too early to draw a definite conclusion on the benefit or failure of HD-AraC addition. Prospective clinical studies as the ongoing trial of the IELSG are needed to definitely evaluate the role of HD-AraC in a combined modality approach. Better selection criteria and better supportive care might help to improve results.

As the majority of patients with PCNSL are debilitated by the neurologic sequelae of the disease, they are at particular risk for infectious complications following myelosuppressive therapies. Our case series also underlines that patients with PCNSL are at risk for fatal thromboembolic events during treatment and neurotoxicity after the end of treatment, reducing overall survival in remission. The increased risk for thromboembolic events in patients with PCNSL is now recognized, while the underlying mechanisms remain unclear^16^. As thromboembolic events can be present already at diagnosis, they do not appear to be treatment-induced or to be dependent on the performance status of the patient or to correlate to immobilization. Analysis of hereditary factors for thrombophilia did not help to identify patients at risk for thromboembolism. We now perform prophylaxis in all patients with low molecular weight heparin. It is unclear whether this will have an impact on the incidence of thromboembolic events as these often are present at diagnosis. In our series, 4 of 9 thromboembolic events were registered at diagnosis before starting cytotoxic therapy. In addition, risks of anticoagulation in patients with brain lesions have to be borne in mind.

Neurotoxicity was the reason for death in 4 patients, all older than 60 years. Neurotoxicity is attributed to WBRT, and patients aged over 60 years are at increased risk^17^. The omission of WBRT form treatment programs however reduces the disease control. Less toxic and more targeted therapies are needed to improve outcome of patients with PCNSL in the future. Examples for such attempts are the addition of monoclonal antibodies, braking/interrupting the brain barrier with mannitol, or intensified therapies supported with peripheral blood stem cell support^18^.

## Figures and Tables

**Table 1.** Patient characteristics

	**total (n=41)**	**HD-MTX (n=19)**	**HD-MTX/AraC (n=22)**

**Age**			
Median, years	59	64	56
Range, years	31–78	31–78	40–72
> 60 years (%)	44	58	41

**Gender**			
Female	21	8	13
Male	20	11	9

**Surgical procedure**			
Stereotactic biopsy	25	14	11
Tumour resection	12	4	8
Not performed	4	1	3

**Lesions**			
Single	15	8	7
Multiple	26	11	15

**IELSG score:**			
0–1	6	2	4
2–3	21	12	9
4–5	8	2	6
n.a.	6	3	3

**CSF cytology**			
Negative	24	11	13
Positive	6	3	3
Not performed	11	5	6

HD-MTX, high-dose methotrexate; AraC, cytarabine; IELSG, international extra-nodal lymphoma study group; CSF, cerebrospinal fluid. The Fisher’s exact test was used to compare the frequencies of patient’s characteristics according to treatment groups. No significant differences were found.

**Table 2.** Feasibility, Toxicity and Outcome of HD-MTX-based chemotherapy in PCNSL

	**HD-MTX** (n=19)	**HD-MTX/HD-AraC (sequential)** (n=13)	**HD-MTX/HD-AraC (combined)** (n=9)
**Renal toxicity** (grade 3/4)	0/0 (0%)	0/0 (0%)	1/0 (11%)
**Liver toxicity** (grade 3/4)	2/1 (16%)	1/0 (8%)	1/0 (11%)
**Anemia** (grade 3/4)	2/1 (16%)	2/0 (15%)	3/1 (44%)
**Thrombocytopenia** (grade 3/4)	2/6 (42 %)	0/5 (38%)	0/5 (56%)
**Neutropenia** (grade 3/4)	1/7 (42%)	0/9 (69%)	0/8 (89%)
**Infections** (grade 3/4)	3/3 (32%)	5/3 (62%)	5/2 (78%)
**Pts who completed chemotherapy as planned**	15 (79%)	9 (69%)	4 (44%)
**Reasons for change of therapy**	Renal tox. 2 Liver tox. 2	Renal tox. 1 Infections 2 Progression 1	Renal tox. 1 Infections 4
**Toxic Death**	3 (16%)	1 (8%)	3 (33%)
**Complete Remission**	12/16 (75%)	7/12 (58 %)	4/6 (67%)

**Table 3.** Causes of Death

**Causes of death**	**Total (n=31)**	**During Therapy (n=10)**	**Following Therapy (n=21)**

**Disease**	16	3	13

**Complications**	11	7	4
**Infectious**	4	3	1
**Thrombembolic**	3	3	0
**Neurologic**	4	1	3

**Other**	4	0	4
**Secondary tumors**	2		2
**Myocardial infarction**	1		1
**Accident**	1		1
